# Conceptualizing Indigenous Human–Animal Relationships in Aotearoa New Zealand: An Ethical Perspective

**DOI:** 10.3390/ani11102899

**Published:** 2021-10-06

**Authors:** Jordan Woodhouse, Anna Carr, Nicola Liebergreen, Lynley Anderson, Ngaio J. Beausoleil, Gosia Zobel, Mike King

**Affiliations:** 1 Bioethics Centre, University of Otago, Dunedin 9016, New Zealand; Jordan.Woodhouse1998@gmail.com (J.W.); nicola.liebergreen@otago.ac.nz (N.L.); lynley.anderson@otago.ac.nz (L.A.); mike.king@otago.ac.nz (M.K.); 2 Department of Tourism, University of Otago, Dunedin 9016, New Zealand; anna.carr@otago.ac.nz; 3 Animal Welfare Science and Bioethics Centre, School of Veterinary Science, Massey University, Private Bag 11-222, Palmerston North 4442, New Zealand; N.J.Beausoleil@massey.ac.nz; 4 Animal Welfare Team, AgResearch Ltd., Ruakura Research Centre, 10 Bisley Road, Hamilton 3214, New Zealand

**Keywords:** indigenous people, Māori, animal welfare, animal ethics, kaitiakitanga, te ao Māori, mauri, wairua, spiritual health, ethics, value

## Abstract

**Simple Summary:**

This article considers ethical views concerning animals of research participants working in animal tourism and conservation who identify as Māori, the indigenous people of Aotearoa New Zealand (hereafter New Zealand). Field work interviews and discussions revealed views about the environment and about the spirit and spiritual connection of people, animals and nature. Understanding the views held by Māori people is important in New Zealand, as it is in any society with an indigenous people, but especially because of Te Tiriti ō Waitangi, one of the founding documents of New Zealand. This partnership agreement between Māori and the British Crown requires and supports a greater understanding of Māori knowledge and culture and accounting for this in our ethical and legal thinking. Our results show that there are factors that the Māori participants consider integral to animal care and management that are different from standard Western views and that it is necessary to reshape how the relationships between humans and animals are considered. We offer ways in which these ethical views of local indigenous community members may be included in policy and laws relevant to animal welfare.

**Abstract:**

This article considers the complexity and diversity of ethical concepts and beliefs held by Māori, the indigenous people of Aotearoa New Zealand (hereafter New Zealand), relating to animals. A combination of interviews and focus group discussions were conducted with individuals who identify as Māori and were working with wildlife, primarily in an eco-tourism and conservation context. Two main themes emerged from the data: ethical concepts relating to the environment, and concepts relating to the spiritual relationships between people, animals and the environment. These findings highlight that the connections between humans and animals through a Māori lens are nuanced in ways not typically accounted for in Western philosophy. This is of particular importance because of the extent to which standard Western thought is embodied in law and policy related to human treatment of animals and the environment. In New Zealand, relationships and partnerships are informed by Te Tiriti ō Waitangi, one of New Zealand’s founding documents. Where these partnerships include activities and environments involving human–animal interaction, policy and legislation should account for Māori knowledge, and diverse of thought among different hapū (tribal groups). We conclude by exploring ways of including Māori ethical concepts around animals in general, and wild animals in particular, in law and policy, providing a case study relevant to other bicultural or multicultural societies.

## 1. Introduction

Animal welfare and human–animal relationships have evolved from concepts discussed by philosophers, ecologists and ethologists to topics of public debate [1]. Māori (the indigenous people of Aotearoa New Zealand) are a case in point of this, developing their own knowledge and values (mātauranga Māori) about animals and their relationships to people. Accompanying this, there is growing demand and support for giving greater respect to the knowledge and values of indigenous peoples across the world [2]. The relationship between mātauranga Māori and animal welfare may be one that is distinct from that found in typical Western thought. This article will cover a case study from Aotearoa New Zealand (hereafter New Zealand) and how mātauranga Māori might explain and progress animal welfare and human–animal relationships. The data contained within this article will be of specific use within a New Zealand context but may find parallels with other indigenous cultures around the world particularly those in the Pacific islands.

Within New Zealand, animals play a considerable role in both society and the national economy. Dairy farming and other agricultural industries contribute significantly to the New Zealand economy, as does the tourism industry, much of which depends on the country’s native wildlife to attract overseas visitors [3,4]. Animals can be affected both positively and negatively by these activities, and they and other practices are increasingly driven by values-based decision-making. To date, frameworks and values of Western origin have dominated animal welfare discourse (e.g., models such as the Five Domains and three circles approach) and this is reflected in policy in New Zealand, as elsewhere in the Western world [5,6].

New Zealand is a society that is committed to biculturalism as underpinned by Te Tiriti ō Waitangi, one of the founding documents of New Zealand signed in 1840 between the British Crown and more than 500 Māori chiefs [7]. Māori identify through whakapapa (genealogy, lineage or descent) from their whanau (family) to their hapū, and iwi affiliations. The term Māori is used to refer to the indigenous people of New Zealand collectively. Hapū refers to kinship group, clan, tribe, subtribe or groupings of extended families. Iwi refers to a collective of hapū forming an extended kinship group, or tribe. It often refers to a large group of people associated with a territory, descended from a common ancestor.

Like the other aspects of mātauranga Māori, the Māori language, te reo Māori, is nuanced and complex. There are many concepts that te reo Māori refers to that are unique and have great importance to the way Māori perceive and understand the world. These concepts are such that it is beyond the scope of this article to provide complete English language definitions of all terms. The limited definitions used in this paper relate to the material discussed herein and are not intended to encompass all aspects of the concepts they refer to.

This Treaty means that there is a need for policy and ethical frameworks that are informed by, or arise from, mātauranga Māori [8]. As part of the framework of New Zealand society it gives political, legal and ethical reasons to understand Māori culture and tradition particularly within specific tribal contexts. Māori settled in New Zealand centuries before Europeans and have a distinctive culture, language, knowledge system, and traditions. This includes a well-developed system of laws, values, beliefs and practices that have developed over time and are deeply embedded in the social context, this is known as tikanga Māori [9]. Tikanga Māori has implications for both what we may consider to be the right way to live and be, and with regard to animals and their state of being, for example what it means for an animal to live a good life [9,10,11].

Given that both animal–human relationships and Māori culture are important to New Zealand, it is crucial for relationships with animals to be understood from a Māori perspective in addition to the currently dominant Western views; the latter have long been the default in animal management contexts particularly within agriculture and fisheries. Ethical consideration of animals can operate in many ways. One can consider animals from various perspectives, at one end of the spectrum animals can be viewed as a species, a population, or a herd, or conversely on the other end of the spectrum, they can be individuals. There is a wide array of literature and research regarding Māori understandings of, and relationships with, the natural world and associated resources, which includes animals. Much of the literature does not have a specific focus on animals as individuals, but instead focuses on them at the level of populations or species [12,13,14,15,16].

A central element of a Māori understanding of the natural world is that humans are a part of it, not in control of it. The whakapapa relationship between Māori and the environment is one that ties them deeply to it, further it establishes that neither animals nor the rest of the natural world, a category that includes humans, exist for the purpose of being exploited and extracted for human use.

Two concepts considered integral to Māori understandings of and relationships with the environment are kaitiakitanga and mana whenua. Kaitiakitanga may be understood as guardianship or stewardship but also encompasses concepts such as resource management stressing the balance of human, material, and non-material elements. Mana whenua is the ultimate and paramount power and authority regarding a territory of land (whenua), derived from the gods. Local whānau and hapū hold mana whenua responsibilities. This transcends legal ownership, and obliges them to manage and protect the whenua in their territories. Both concepts are generally not discussed within policy settings, nor academically, in relation to obligations to individual animals, but rather in terms of species, populations of animals, the environment or the ecosystem as a whole [10,12]. For example, when eel (tuna—*Anguilla dieffenbachii* and *Anguilla australis*, species of freshwater eel indigenous to New Zealand) are mentioned with reference to kaitiakitanga it is most commonly in discussions about the body of water they live in, and the population of tuna as a whole [17,18]. Such holistic views of the environment are reflective of a concern for overall environmental well-being as opposed to imbalances that may arise from a focus on singular species or entities.

Likewise, there are a number of academic accounts of Māori health, an important component of animal welfare [19], but not specifically of the health of animals. Both Te Whare Tapa Whā, the four cornerstones model of Māori health and well-being, and Te Wheke, the octopus model of Māori health and well-being, have a human focus [20,21]. However, some of the elements in these accounts may suggest applications to animal welfare from a Māori perspective, insofar as, from that perspective, humans and animals may be similar for the purposes of health and welfare.

Two valuable accounts of welfare that find common use within animal-focused industries are the Five Domains model and the three circles model [5,6,19]. These models serve as valuable tools for assessing the welfare of animals, especially in ways that can be measured or contrasted against one another. The Five Domains model consists of four physical domains relating to the animal’s physical state and interactions with its environment and other animals (nutrition, environment, physical health, and behavioral interaction) and one mental domain. Observable evidence of impacts collated in the four physical domains informs understanding of positive and negative affective states in the mental domain that are considered most relevant to the animal’s overall welfare state [22]. The Five Domains model itself does not provide criteria for what is a good or acceptable life but is predicated on the notion that individual animal’s affective experiences matter to them and thus are of key importance for understanding their welfare states [23].

The three circles model consists of three overlapping circles akin to a Venn diagram [5]. The three circles are physical health and functioning, affective states and natural living, which represent fundamentally different value positions about what a good life for animals consists of. This model proposes that welfare science cannot resolve these differences, and any assessment of animal welfare must consider all three (like the three circles model, the Five Domains model implicitly considers the physical health and functioning, and natural living states of the animal but only in so far as they influence affective states). Whether these accounts would, and could, be compatible with a Māori understanding of animal welfare is an open question which this research aims to address.

To elucidate Māori perspectives with regard to animal welfare and human–animal relationships, we conducted a series of interviews and focus groups. Direct conversations with Māori individuals and groups from both the tourism and conservation sectors provided insight into how animals are viewed. The context and detail garnered from these conversations may be used to develop an understanding of the relationships between Māori and animals, with implications for ethics and welfare.

The aim of this research is to examine the views of Māori with regard to human–animal relationships and use this data to contribute to an articulation of Māori views about human–animal relationships and animal welfare and how these views may align with commonly accepted animal welfare thought of Western origin. We should note that any reference to a body of thought such as “Western” and “Māori” or “indigenous” risks mischaracterizing it as homogenous, when each reveal diverse theories, concepts, and values. In all cases where we use these terms, we are referring to thought arising from people who are of these origins, or which derives from established concepts with that origin, and do not mean to make or imply any broader generalizations about Western, Māori or indigenous thought.

## 2. Materials and Methods

### 2.1. Rationale for Research Approach

This research is grounded in a constructivist epistemological position; it aims to gain knowledge about the world through others experiences and beliefs, in this case the relationship between Māori and animals [24]. Alongside the existing academic literature, this method is the most likely to develop a nuanced understanding of animal welfare; it is influenced by detailed Māori experiences expressed through participants’ narratives.

We have sought to limit the way our own knowledge may influence these data through the use of informed grounded theory in the data analysis [25], but we acknowledge that this data cannot be truly objective. The narratives have been gathered and interpreted through an academic research process. Nonetheless, we note that all researchers reflected on this potential subjectivity throughout the data collection and analysis process. In order to do this, Informed Grounded Theory methodology was used and the research was conducted in a way that aimed to respect and nurture the unique cultural identity of Māori.

### 2.2. Study Design

This research project has been informed by both written and kanohi ki te kanohi (face to face) consultation with the Ngāi Tahu (the principal iwi (tribe) of the South Island of New Zealand, also known as Kāi Tahu) Research Consultation Committee, at the University of Otago. Ethical approval was gained through the University of Otago Human Ethics Committee, Application 20/012. The focus group and interviews were conducted between the dates of 19 June 2020 and 26 June 2020 with individuals from Māori backgrounds who were working, or had recently worked, in wild animal tourism and conservation.

Members of the focus groups and interview participants were recruited via snowball and convenience sampling. Individuals known to the researchers as having extensive interactions with animals were approached, as they were deemed to be ideal participants in the study.

Inclusion criteria were: (1) the participant must identify as having Māori ancestry, and (2) the participants must have significant interactions with animals or other ways of relating to animals that have given them reason to reflect on animal welfare. Exclusion criteria included non-Māori, those living outside New Zealand; those under 16 years of age and those who were unwilling to have their views recorded and potentially published.

Six participants contributed to our research over two individual interviews and one focus group discussion undertaken in personal meetings that enabled engagement that was kanohi ki te kanohi. These participants whakapapa (trace their ancestry) to the Kāi Tahu, Kāti Māmoe, Wāitaha, Rāpuwai or Kāti Hāwea iwi and hapū.

### 2.3. Interview Structure

The interviews and focus group lasted between 60 and 90 min and followed semi-structured questions to guide the narratives around participants’ views and beliefs regarding animal welfare and their relationship with animals. This method allowed for discussion to develop organically and explore detailed understandings. The focus of the researchers in the interviews and focus group was upon an individual’s personal interactions with animals in professional workspaces and their reflections on individual beliefs or philosophies. This enabled participants to express and reflect on their views and understandings of animal welfare and their relationships with animals with insights into whether, and in what form, their understandings were informed by te ao Māori (the Māori worldview), or mātauranga Māori. The interviews were digitally audio recorded and transcribed.

### 2.4. Data Analysis

Thematic analysis of the transcripts was conducted using NVivo qualitative analysis software. Informed Grounded Theory (IGT) was used to analyze the transcribed data. IGT is a data analysis methodology based on the principle of using Grounded Theory (GT) methods informed by the existing research literature and theoretical frameworks [25]. GT methods are designed for investigating areas where there has been previously little to no research performed (e.g., the nature of the relationship between te ao Māori and the welfare experiences of individual animals). IGT expands on these basics by incorporating the literature research through a neutral theoretical stance that allows for clear conclusions to be drawn from the obtained data without allowing previously known theories to taint or obscure these conclusions.

### 2.5. Positioning the Researcher

As a multicultural research team, many of whom do not have Māori ancestry, there are a bevy of ethical considerations that must be taken into account [26]. There have historically been a number of cases where indigenous cultures were exploited or misrepresented through research, and this has understandably fostered a sense of mistrust towards prospective researchers in some cases [27]. One of the research team members is of Māori descent and guided our research process with particular focus on issues of tikanga to ensure cultural respect. Every effort was made to ensure that the data obtained through this research were both accurate and appreciative of the complexities of the rich and detailed Māori worldview.

Efforts were made to translate concepts between Māori and English as accurately as possible, however, there may still be nuanced elements of Māori concepts that may be lost in translation and cannot be fully explained in English—they can only be truly expressed in te reo Māori (Māori language). None of the research participants were native speakers of te reo Māori but all were at various stages of learning. The literature reviewed for the project was also written in English and thus may suffer from similar issues. Additionally, as an oral tradition some Māori knowledge was likely not available for us to access.

## 3. Results

Analysis of the transcripts obtained over the course of the interviews and focus group discussions conducted revealed several key themes that were mentioned across multiple transcripts. Three themes identified in our analysis were kaitiakitanga, mauri (the life force or vital essence of a thing), and spiritual connections.

### 3.1. Kaitiakitanga and the Natural State of Things

Participants expressed strong support for the protection and promotion of indigenous New Zealand species, as well as efforts that enable this through habitat preservation. Participants saw an integral relationship between animals and the environment. One participant who worked in penguin conservation said the following:

*“Wearing my kaitiaki* [guardian] *hat looking at the blue penguin I use them as an indicator species because they don’t travel too far. They indicate to me how healthy the marine environment is.”*

This participant viewed individual animals as elements relating to a greater environment and that this link was important when viewing things from a kaitiakitanga perspective. There was a common notion amongst the participants that improving the health of the environment would improve the welfare of animals within it.

When discussing conservation, there was a significant focus on pest control and the removal of invasive species. Emphasis was placed on the importance of a natural environment, one that was ‘untainted’ by both invasive species and destructive human interaction. A trend among participants was to advocate for a strategy of limited intervention with indigenous species whenever possible, with the exception of ensuring that their environments remained free from invasive predators and other outside influences. As one participant said “*Human intervention is a big mistake*”, this highlighted the view that human intervention should be avoided unless there is no other acceptable alternative.

Action was justified to encourage the survival of particularly threatened species, but any intervention would be limited as much as possible. Overall, a natural environment was seen as something desirable, at least with regard to those animals not equipped to deal with the foreign elements that may have been introduced.

A participant who had ties to native bird conservation placed value on providing an environment where animals could flourish free of ‘unnatural’ predators (e.g., rats, stoats, possums and other creatures that had been introduced by humans). In describing some of the sanctuaries where she worked, she said:

*“We don’t have rabbits, goats, pigs, stoats, ferrets. We don’t have any of the mustelid family. They are killing machines.”*

She stressed that these introduced animals were eliminated due to the damage they do to an environment not evolved to account for them. A priority was placed on maintaining what could be called the ‘purity’ of the natural world, keeping it free of foreign elements that might disturb or damage it.

This went further than merely keeping introduced or invasive animals out of the environment, however. It extended even into the interactions between humans and native animals. One of our conservation-focused participants, when asked about their policy with injured animals, said:

*“The natural life should not be interfered with too much; sometimes we cause more trouble than we mean to… What would happen if we weren’t there? If you remove us from the equation, they usually can look after themselves. It is whoever is the fittest. When there are three bright orange beaks coming out of the nest, the parents just aim for the brightest color that they can put the food into, and those birds will trample over each other to get to the top. It is the strongest one that will survive, and that is what makes the next generation strong because only the strong survive. It is a hard lesson for some people to learn.”*

Participants emphasized the importance of native animal species living in a natural environment and engaging in natural behaviors, including predation. It was said that:

*“If a bird falls out of the nest and a weka* [Weka—Gallirallus australis, a flightless bird endemic to New Zealand] *comes along and takes off with it, it’s part of the natural world; it’s our Serengeti”.*

This is seen as beneficial for animals, allowing for the strongest to survive and contribute to the next generation, thus allowing them to be strong as well. Human interference with this process was seen by one participant as detrimental and should be limited when possible. This relates to the value placed on preserving species for the future; what is beneficial for animals is not directly beneficial for the individual animal but rather for the survival and flourishing of the species as a whole.

The interviews also explored the notion of the value of species. The idea that different species of animals are valued differently and thus the welfare of the less valuable could be sacrificed to further that of the other. This was most apparent between the treatment of invasive species such as rats or stoats and the treatment of indigenous wildlife. Invasive pest and predator species were universally valued less than the indigenous wildlife, with campaigns to remove them from, or curtail their actions within, protected areas being encouraged and supported.

*“We are doing all this work to protect our native species. Long term, we will see cats being an inside pet. You will have to be a registered breeder to be able to move them and that process will be so arduous that there aren’t many breeders, and we would see the population drop to about 5% over about ten years.”*

This is in direct contrast to indigenous animals, where engaging in their natural behaviors was encouraged and considered important to their welfare.

There is a greater element to this, however, than merely the inherent value a species has through its status as native to New Zealand. There is also the value that is given to species based on what they are able to provide or do for us. A participant, when questioned about why they felt the conservation of muttonbird (titi—*Puffinus griseus*, also known as the sooty shearwater or muttonbird) was important, said:

*“I think it is as simple as I like eating them, and it is no different than a European farmer for thousands of years ensuring that sheep are around”.*

So, it is not only the inherent native status of a species that may cause it to be valued but the use that can be obtained from that species. Animals that can be harvested or can provide some kind of service have a value of their own. As one participant said.

*“Everybody wants to see the wildlife in this pristine environment. It is unique and people just love to see the freedom. We are the visitors there. The wildlife and the forest are paramount, and we are just privileged to live in that environment.”*

The value of species and individual animals can stem from the relationship individual members of the species have with humans. This value may be derived from direct interaction with animals or from less tangible benefits gained from their presence.

The considerations intertwined in kaitiakitanga also play a role in how the welfare of animals might be thought about or prioritized. Many participants reported being directly involved in conservation efforts in order to encourage the survival of indigenous species. They played a direct role in the preservation of native species within New Zealand. One participant said that the conservation work they were doing today was especially important for future generations and the relationship they would have with the environment.

*“[I want] for them to tell the story of success, hand those stories down. That is how we work, that is how I work. Hand those stories down. They are beautiful stories for our mokopuna’s* [grandchild or grandchildren]. *I grew up with my grandparents. […] My mum would say to me today it is not about you; it is about the mokopuna. It is about us doing our best for our mokopuna that we can.”*

The above comment highlights that for this participant, the impetus behind conservation efforts and the values of guardianship that kaitiakitanga encourages is to allow mokopuna and future generations to experience the environment that is available now, ideally in a better condition than it is currently. This participant held views that there is value to be found in interacting with the environment and the indigenous wildlife that live within it. Further, it suggests that this value may be thought of as valuable beyond their personal experiences. It can be seen as something that should be valued by others in the future. The value of preserving the environment lies not only in one’s personal interactions with that environment, but the interactions one’s descendants will have down the line.

Another participant considered that the land itself has intrinsic value, regardless of whether humans are present or not.

*“I think the humans will die off before the animals do. We have to ensure that our ethos that comes from ourselves as Kai Tāhu is to leave the whenua* [the land] *in a better condition than when the responsibility was passed to us. That includes everything that is living upon it.”*

This goes beyond the perspective that wildlife should be preserved for future generations, it suggests that value may not only be found in the environment through experiencing it, but that there is some inherent value in leaving it in a better condition than it was before. This leads into one of the other major themes we identified, the mauri of the environment.

### 3.2. The Mauri of Things

Mauri was another concept that underpinned many of the things we discussed. One participant described mauri as the following:

*“It is a lifeforce, an essence of life. Another way of thinking is the spirit in te ao Māori is the wairua, and that is something beyond death and can interact with the afterlife, but the mauri is something that is “life”, and if it dies, it dies.”*

Mauri was seen by participants as an essence that was present in all living things and in the world around them. There is inherent value in mauri, and enhancing the mauri of a place is something to be encouraged and desired. To some participants mauri was not seen as something that dissipated when a living creature died, but rather something that changed forms and remained within the environment. Although the mauri of the creature is gone it is also in some ways passed on. One participant described this as a cycle:

*“When I think of Ki uta ki tai which is mountains to the sea, that really is about mauri because you have that rain that comes onto the mountains, eventually coming down the rivers, going out into the ocean and then going back up onto the mountains. It is a closed circle, and you are impacting on that mauri by discharging things into that environment or, when you are harvesting things, killing more and leaving that lying in the ground and contaminating the ground or contaminating the waters.”*

This relates to the value that is placed on animals living in a natural environment; by allowing them to live in their natural state, their mauri with is not impacted upon as it might be if there was regular intervention. The elimination of pest and predator species also works towards this goal, limiting their impact upon the mauri of the environment and allowing the mauri to flourish as it would without their presence.

Due to the interconnected relationship between wildlife and the environment, the acknowledgment of mauri within the environment can be seen as directly beneficial to the welfare of animals within that environment. When discussing involving themselves in an advocacy space for wildlife one participant expressed the following:

*“If we have enough mauri within ourselves to come to those tables, then that will then come back to protecting our wildlife.”*

This highlights a belief that if the life force within them is strong enough to drive them to advocate for the land and the wildlife, then that mauri works to restore and improve the mauri of the land.

Furthermore, the mauri of the environment can allow it to be seen as a living entity in its own right. In our discussions, we heard many references to the idea that the land itself was alive and needed to have its own voice. One participant spoke of their personal experiences of advocating on behalf of the environment in the Environment Court, a New Zealand court that hears issues arising under the Resource Management Act 1991 [28]:

*“I have stood up in Environment Court and talked on behalf of the trees, because the trees aren’t considered an entity…”*

This ties into the idea that Māori may act as representatives for the lands for which they hold mana whenua; they act not as owners, but as advocates for entities that play an important role in their lives and beliefs.

*“This has happened only in the last two decades through people doing it, standing up and saying ‘I put this submission on behalf of the trees that are going to be affected by the activity involved’…”*

Thus, the mauri of things is thought not to be separable; it is an intrinsic quality that must be respected, acknowledged, and advocated for. This connection to mauri is one that exists on a spiritual level for Māori, informing the final major theme about the spiritual connections between things.

### 3.3. Spiritual Connections and Whakapapa

The spiritual and familial connections between participants and animals was another theme evident in the research. Two major categories were present within this theme: the spiritual and familial connections between participants and the land, and the spiritual and familial connections between participants and wildlife.

#### 3.3.1. The Land

Participants said that the land has tremendous spiritual significance. The health and management of the land were considered to be of great importance. This, in turn, was seen to be directly related to being spiritually connected to the wildlife that lived on that land. Caring for the wildlife that lived on the land was seen as the same as caring for the land itself. When asked why it was important to continue improving the welfare of one of their taonga species, one participant said:

*“Because it is a part of who we are, and it is really important for us as Māori to keep connecting to the land.”*

This participant evidently saw no distinction between looking after the land and looking after the species that live on it. The relationship with nature was holistic—by being spiritually connected to the land, they are spiritually connected to the wildlife that lives there. The land they consider to be their ancestral home is a part of them, with deep connections that go back many generations.

*“She was brought up in that lighthouse, and her whenua* [placenta—also used to refer to the land or domain] *is buried underground. My grandmother’s whenua was buried underground.”*

The generations that have lived on traditional lands have left physical and spiritual impressions that are significant to their descendants. This is reflected in pepeha, a way of introducing oneself in Māori in a process that shares connections to people and places that are important to you and where you come from, and other aspects of Māori practices where they express the connection to the land, particularly landscape features that are viewed as ancestors through whakapapa.

*“We understand in that whakapapa […] the land and the animals are our ancestors, and, in that relationship, we are subservient to the land and the animals.”*

The familial relationship between participants and the land is one where they consider the land to be part of their ancestry and feel the same obligations to it that they feel to their ancestors. Participants said that those who have been denied access to this land feel they can replenish well-being and heal by rebuilding their connections to it.

#### 3.3.2. The Animals

A connection to animals was also reported through whakapapa, where some animals connected through common ancestors of Māori. In a discussion about management rights being restored, one participant said:

*“We have been cut off from our land and our taonga* [treasured] *species* *for a long time.”*

The context around this is again holistic—the same familial obligation that is felt towards the land extends to the animals as well. Being separated from those elements may be just as harmful as being separated from one’s human family and ancestors according to several Māori participants’ understandings.

As mentioned before, the spiritual connection to wildlife is deeply intertwined with the spiritual connection to the land. Often, connecting with one is also connecting with the other.

*“I believe that the albatross and the titi and the penguins, so ko tangata toroa, ko tangata titi, ko tangata kororā are waiting for us to come back home, come back to here [to the land] and look after them properly.”*

This view is indicative of the value of kaitiakitanga where those who have spiritual and familial ties to the land can truly be the kaitiaki who look after the natural inhabitants. That without these ties, there is something that may be missing that can contribute to the welfare of the wildlife. When the healing that can be gained from the land and wildlife was discussed, it was asked if the wildlife can benefit from this healing as well.

*“Yes, I do think so. One of the things I have been noticing is that they need more protection and advocacy space. The protection of their fishing grounds, the protection of their environment in things like the Port Otago Mana Whenua consultation group and things like that. If we have enough mauri within ourselves to come to those tables, then that will then come back to protecting our wildlife.”*

An almost symbiotic connection is suggested, where the participants benefit from the presence of the land and the wildlife and the wildlife benefit from the kaitiaki who advocate and speak on the behalf of the natural environment and animals within.

## 4. Discussion

The views of participants covered a range of different concepts when discussing the ethical relationship between Māori and animals. In this paper those views will now be discussed according to two themes—the natural world or the spiritual world—noting that both worlds are seen as interconnected according to Māori tikanga and practices such as wāhi tapu (a place sacred to Māori in the traditional, spiritual, religious, ritual or mythological sense). Each of these themes has its own sub-themes. The natural world refers to elements of kaitiakitanga and the natural environment, while the spiritual world refers to the relationship between animals and Māori, the spiritual health of animals, and the concept of mauri.

### 4.1. The Natural World

The importance of the natural world was a common topic within the discussions. The natural world held great significance to participants as their ancestral lands containing various natural taonga, to whom they have kaitiaki and genealogical relationships, associated with cultural values and tikanga.

#### 4.1.1. Kaitiakitanga

As previously mentioned, one of the most prevalent themes distinguishing the relationship between Māori and animals was the concept of kaitiakitanga. Kaitiakitanga is not simply a set of behaviors or actions one must perform, rather it extends to a philosophy or way of thinking about the natural world (human and non-human, living and non-living) that is greater than this [12,13]. This obligation of guardianship and caring for the environment was a core concept that participants expressed when discussing their views and goals for the environment, particularly those participants with connections to conservation efforts. They stressed the importance of caring for the environment and working to keep it healthy and flourishing.

Given the environmental focus of kaitiakitanga, which tends to emphasize groups, populations, species, ecosystems and includes the non-living elements of the environment as well as the living, it can seem that kaitiakitanga does not focus on the welfare of individual animals as such. However, this is arguably a mischaracterization that overlooks the holism of te ao Māori; from a Māori perspective, individual welfare may not be achievable in isolation from others. Patterson stated that *“the welfare of the whole depends upon that of each individual; the welfare of each individual depends on that of the whole”*, clearly establishing that welfare is an interconnected principle [15]. It is dependent on other related parties also having a high degree of welfare and contributing to the welfare of others in turn.

This seems to align with the views of participants; those with commitments to conservation emphasized the importance of the population as a whole over individual animals, at points advocating against intervening to improve the situation of individuals as this was perceived ultimately to be harmful. Kaitiakitanga has a holistic focus rather than an individualistic one, and if welfare is also conceived of holistically, it may also be provided for.

Kaitiakitanga includes recognition that many individual animals have their own mana (power or status; mana is a supernatural force accompanied by status and power) and mauri—there are numerous accounts of Māori traditions where animals are taonga species and may take on the role of kaitiaki or guardians for humans or natural areas. For example, Schwimmer (1963) discussed the role of individual animals inhabited by atua (gods) that were guardians of Ngāti Wai iwi in the Northland region [29]. As atua, these animals have mana (and in Whangaruru are referred to using the term mana) which Schwimmer explains expresses the belief within Ngāti Wai that these animals both have mana, and are also the source of the mana that people have.

Kaitiakitanga exists at a nexus of the spirit, the environment and the human [12]. A tendency we observed within our discussions was that, from a Māori perspective, the health of the wildlife in an area would often be treated synonymously with the health of the land. Some participants regarded improving the health of the land and improving the health of the wildlife upon that land as essentially synonymous. This has specific implications for the way Māori may view animal welfare as it suggests that from their perspective, work that improves the health of the land feeds back into improving the health of the animals, and consequently animal welfare.

The Māori worldview is primarily holistic [30]. This, however, highlights one of the intricacies of kaitiakitanga as a concept when applied to something such as animal welfare. Kaitiakitanga is a traditional and sustainable approach to environmental and resource management. As exemplified in our findings, acts of kaitiakitanga are holistic in character. It can justify care for populations of animals and species, while at the same time advocate for the care of individual animals within them. Because this care is holistic, focusing attention at the group level will benefit both the group and the individuals within it; in turn, focusing on any individual will also benefit the group, and ecosystem.

From a contemporary Western perspective, welfare is not often conceived of holistically. Although animal welfare may be assessed on a population-wide level, this is not generally considered the primary focus of welfare. Welfare, as defined by Fraser et al. (1997), is something individuals have, and this may be aggregated to determine the population welfare [19]. While this links individual and population-level welfare, it is possible, from this view, for a population to have a good total or average welfare, while some individuals within the population have poor welfare. In fact, the welfare of the population may, over time, be improved by some individuals faring poorly, since this means there will be selection pressure against them and any disadvantageous traits they may possess, in favor of fitter individuals. This is different from a kaitiakitanga approach, which appears to emphasize a unity of individual and population levels of welfare, rather than them being two separate and different things that can be in conflict with each other.

#### 4.1.2. The Natural Environment

The natural environment emerged as an important element of the relationship between Māori and animals. This is in line with the emphasis that Māori ethical concepts put on respecting the natural world. As part of that, several participants advocated limiting human interference with the natural way of the world. Some participants suggested that non-intervention is important for species to allow them to develop naturally and that by interfering in their development through actions such as medical intervention, the ability of future generations to survive could be negatively impacted. It would appear that the ability to sustain and provide for oneself is considered important to the welfare of an animal from a Māori perspective. This may be similar to how adaptations being used and natural behaviors being expressed is important within a natural living orientation to animal welfare [19,31].

Interestingly, this non-intervention was disregarded in cases where the survival of an indigenous species was sufficiently threatened, suggesting that while it may be important to welfare, it is of lesser importance than the survival of the valuable population or species. Some participants stated that interactions that were harmonious with and not disruptive to those animals’ own behaviors and environment were permissible, therefore non-intervention was perhaps advocated primarily in preference to disharmonious intervention that could otherwise occur. This is, in some ways, a mirror of Māori traditions that focus on achieving this objective in other contexts, such as the traditional Māori method of selecting trees to construct a waka (traditional canoe) through careful and respectful ritual; this is demonstrated in the tale of Rata and his canoe [30].

It should be noted however, that the term ‘natural’ can be interpreted in many ways. There is the commonly accepted meaning that refers to the environment absent of human constructs, the portion of the world that has not been created by humans or significantly redeveloped by them. This is the view expressed in nature-culture dualism, where ‘culture’ refers to all human artifacts (e.g., cars, buildings, tracks through the forests made by people) and ‘nature’ to everything else not produced through human agency [32]. However, among participants we noticed a further, or alternative, division of what was ‘natural’ and what was ‘unnatural’. There was a perception that the category of ‘unnatural’ extended beyond things merely created by humans, to things such as species that humans had played a role in introducing. With regard to the New Zealand environment the ‘natural’ environment was thought to consist of those plants and animals native to New Zealand prior to colonization by Pākehā (Non-Māori, foreigner, New Zealand European).

In particular, a distinction was perceived to exist between native and non-native species, with the former natural, while others were not. Those species such as rats, stoats and possums were thought to be ‘unnatural’ in that the native environment of New Zealand was not adapted to them. Removing them from areas within New Zealand and preventing them from preying on native wildlife was seen as protecting or restoring the ‘natural’ environment.

### 4.2. The Spiritual World

The spiritual world is ingrained in Māori thought and tradition [33,34,35]. Humans, animals, plants and all living entities have a wairua, a spirit or soul that persists beyond death [35]. Interviews with participants did not involve discussion about whether there was a spiritual world or how it interacted with the material world; for some it was expressed as integral to their understanding of the world.

#### 4.2.1. Connections between Māori and Animals

At the forefront of Māori relationships to animals is the wider setting of the surrounding landscape, as one participant stated: “*…it is a part of who we are, and it is really important for us as Māori to keep connecting to the land.”* This is recurrent in Māori creation tradition, te orokohanga o te ao (the beginning of the world), that tells the origins of the land. The land itself is an ancestor, known as Papatūānuku (the mother earth in the Māori account of creation, the first female element of the universe and the giver and sustainer of life. Mother of the first atua—the first gods) in Māori cosmology, one that they are connected to and must honor as they do the human ancestors who have come before them [13].

The recitation of whakapapa, one’s genealogy back across the generations to where one is related to the gods, is a concrete example of how Māori identify as being inseparable from nature. Whakapapa extends beyond genealogy to provide an understanding of how there are many interconnections with the natural world—calling back to the land, traditional gods, ancestors and distant relations. Animals can be thought of as kin, both in the sense that there can be an emotional bond between animals and Māori but also in the sense that some Māori believe that animals are part of their ancestry [10,13,36]. One participant told a story of their mother and grandmother visiting them in the form of an albatross on their mother’s birthday. The emotion the participant shared when recounting this story did much to convey the significance the experience had to them and the importance they gave the spiritual relationship they had with both their ancestors and the albatross.

The bonds between Māori, animals and the land are thought to contribute to the welfare of animals, where interconnections mean the health of one influences the health of the others. On one level, this seems common sense, as animals living in an environment that is not healthy seem likely to fare worse than those in a healthy environment. This seemed to extend beyond the physical into the spiritual nature of animals and was deeply intertwined with the land and consequently with Māori who have ancestral ties to the land, as tangata whenua (people born of the land, and who therefore whole hold mana whenua). This is demonstrated in the reference to land in whakapapa where Māori often refer to their hapū or iwi’s mountain and waterway. It was said by a participant that “*when you are connected with the land, I believe they [animals] connect with us*”, this was in the context of spiritually important events taking place between Māori people and animals. This is particularly relevant to taonga species, those species attributed with particular cultural or sacred characteristics or values, which have special importance for Māori.

#### 4.2.2. The Spiritual Health of Animals

Another major theme evident in our research is the emphasis on spiritual health. Spiritual values have incredible potency within Māori culture and traditions and were of visible importance to participants. In his definition of wairua, Barlow (1994) says that Māori understand that all things have a spirit, not merely humans but “birds, animals, fish and even the earth itself” [35].

Finally, Durie (1998) and Pere (1991) conceptualized models of health informed by Māori values and knowledge—Te Whare Tapa Whā and Te Wheke—that strongly emphasize the importance of spirituality to the health and welfare of individuals [20,21,36,37]. Although these are accounts of human health, they may be helpful in understanding animal welfare. Many Western philosophical conceptions of animal welfare can be found to draw from accounts of human welfare, so it is a plausible option for a Māori understanding of animal welfare to do the same. It is already known that the environment is thought to have a form of spiritual health so it is possible that animals on both individual and population levels are also influenced by spiritual health.

#### 4.2.3. Mauri

The concept of mauri, of vital energy, arose frequently in our research. Mauri is a vital part of the very existence of things within te ao Māori [38]. Barlow (1994) defines mauri as what merges the physical and spiritual parts of an entity together [35]. The emphasis on the value of enhancing the mauri of a place was a common sentiment expressed by participants. One participant defined mauri as *“a lifeforce, an essence of life”* and it was a common theme arising within discussions for the mauri of a place or area to be considered important. However, it goes beyond merely being an animating force; mauri makes it possible for a thing to exist within the bounds of its own creation [35]. It is not merely about life but about the essence of a thing, and the enhancement and support of that essence is thought to be a thing to strive for.

Enhancing mauri is most often achieved when restoring the natural state of an environment. Damage to, or contamination of, the environment is damage to or loss of the environment’s mauri [16,39]. When participants discussed the concept, it was with regard to how they might improve the mauri of an area, such as a stretch of coastline or a waterway. Although animals were referenced in these discussions, the focus was on the environment as a whole (including animals) rather than on the animals as individuals. However, as living creatures, the wildlife undisputedly has mauri of its own and is also considered part of the environment in which it lives [13,15]. As noticed by a participant in reference to *“ki uta ki tai”*, there is a cyclical relationship within the environment where mauri interacts with animals, plants and other components. Enhancing the mauri of the environment in such a way that it also enhances the mauri of animals within that environment is valuable and beneficial for them.

Possession of mauri is commonly used as a reason why entities should be respected, but how does this relate to the welfare of animals? One of the interpretations of mauri that Patterson (1998) highlights is that it can be thought of as character or essence in addition to lifeforce [15]. Patterson explains that respecting mauri involves understanding distinctive qualities of creatures (and other things in nature) and respecting them for what they are [15]. Fraser (1997) describes one account of welfare (natural living) that values an animal’s ability to engage in natural behavior and use their natural capabilities [19]. The alignment of these two views is striking.

Both views suggest similarities to the Aristotelian concept of *telos*. *Telos* is the ultimate aim or purpose of a thing. In relation to animal welfare this has been interpreted as the behavioral needs of an animal as a characteristic member of its species (for review of accounts of *telos*, see [40,41]). On this view, one must understand the distinctive behavioral qualities of animals, usually characteristic of members of their species in general, in order to know what a good life for them is, and determine whether they are leading a good life. The opportunity to use and develop characteristic behaviors is constitutive of a good life and any instance of such behaviors contributes intrinsically to the welfare of the animal. For example, a good life for birds is one in which they can fly, unless they are a flightless species. This potential similarity with Aristotelian thought will be revisited in our discussion of ethical implications of this research.

Overall, it is apparent that there are many aspects of Māori thought that provide ways to consider the lives of animals that are distinct from typical Western understandings. The spiritual links between Māori and animals and between animals and the environment could have significant implications for animal welfare practices in New Zealand.

## 5. Conclusions

### 5.1. Implications

#### 5.1.1. Implications for Law and Policy

Māori values play an important role in policy development within New Zealand stemming back to the establishment of the Te Tiriti ō Waitangi [7]. This is reflected to varying degrees in legislation, for example the New Zealand Resource Management Act 1991 explicitly incorporates some Māori concepts, as does the New Zealand Conservation Act 1987 [28,42]. However, the primary piece of animal-centric legislation within New Zealand, the Animal Welfare Act 1999 (AWA) [43] does not have any reference to Māori concepts. This is also the case for other related legislation such as the Wildlife Act 1953 and the National Parks Act 1980 [44,45]. The Marine Mammals Protection Act 1978 has some small mention of Māori consultation but no mention of Māori concepts outside of this [46].

Among participants there was call for the continuation and implementation of certain animal-related policies, such as pest-control policies, from which the natural environments they worked in can benefit. Importantly, through Te Tiriti ō Waitangi settlements the customary uses of animal species are often recognized within resulting Acts of legislation, e.g., Ngāi Tāhu Claims Settlement Act 1998 [47]. Participants were individuals from eco-tourism and conservation backgrounds. They all held similar views regarding government policies that affected their relationship to wildlife, such as those around customary use, protecting native species or controlling the spread of invasive species.

Given the bicultural political foundation of New Zealand, laws relating to the treatment and welfare of animals, such as the Animal Welfare Act 1999, the Wildlife Act 1953, the National Parks Act 1980, the Veterinarians Act 2005, the Biosecurity Act 1993, and the Conservation Act 1987, ought to be reviewed for their inclusion of relevant Māori ethical concepts and values, and the degree to which they accommodate or facilitate Māori approaches to customary use, and animal care, use and management [42,43,44,45,48,49]. Our research has highlighted that there are factors that Māori consider integral to wild animal care and management that are different from the standard Western views. The inclusion of these values within relevant legislation would further the goal of protecting animal welfare, and also support integrating Māori concepts and values into policy and practice. There are also instances where customary use of certain wildlife species occurs, for example kiwi feathers used in raranga (weaving), and tītī (muttonbird) used for kai (food). Legislative reviews and resulting management would ideally not interfere with such customary use without full engagement with those hapū and iwi affected.

#### 5.1.2. Implications for Models of Animal Welfare

The Five Domains model and the three circles model represent a standard Western understanding of welfare and are broadly accepted and used to perform animal welfare assessments in a wide range of areas. The need to account for Māori concepts and allow for practices of tikanga Māori, and indigenous thinking more generally, within such assessments raises the question of how these models might align with Māori or indigenous understandings or be revised to do so.

The Five Domains model does not explicitly include any Māori concepts. Key concepts that may be relevant for inclusion are the wairua and mauri of animals which may correlate with domains within the model. For example, if an animal with weakened mauri or wairua means that it will be unable to express natural behaviors, or be in good health, and/or will experience negative affective states, then this may be accounted for by the model, since these are present in it.

However, the Five Domains model is dependent on the sentience of an animal in order for it to be applied. Its emphasis on affective states as integral to welfare presupposes that animals are capable of experiencing these states. Aspects of mauri or mana however, do not depend on sentience; when an animal dies, parts of its body may be seen to have mana or mauri of their own. Or it may be that non-sentient life has its own mauri and thus welfare. The Five Domains model could not account for these circumstances. Certainly, this does not diminish its usefulness, the majority of cases that require animal welfare considerations likely deal with sentient animals and for this purpose the model is still useful. However, outside of this it may struggle to adapt to a Māori conception of welfare.

The three circles model appears to have similar difficulties as the Five Domains model with regard to spiritual elements of welfare. In order to integrate a Māori view of welfare into the three circles approach, it may be necessary to incorporate elements of spiritual welfare in one or more of the circles. Alternatively, there may be some possible commonalities; the spiritual emphasis on connections to the environment appears to mesh well with the natural living circle. This circle covers most of the functional components of living in one’s natural environment and may also be thought to include the spiritual connections and dependencies an individual might have to the land they live in. Fraser’s (1997) position that welfare has an element of natural behavior to it supports that mauri may be encompassed by this circle [19]. The greatest difficulty lies in the notion of a purely spiritual element of health that is unrelated to the environment, however, the holistic view te ao Māori takes of the world means that this may be avoided through the connections such a state may have to other aspects of health.

There is, therefore, potential for these models of animal welfare to be revised to incorporate Māori ethical concepts. This may smooth the process of introducing such concepts into welfare practice and assessment.

#### 5.1.3. Ethical Implications

There is significant debate about the ethics of the various uses of animals that are the subject of this paper, such as animal tourism [50,51] and farming [52,53,54] including whether and how they should be reformed, or whether they are ethically permissible in any form. All are based on valuing animals instrumentally, while not in all cases precluding the fact that they can also be valued intrinsically (any acceptance that animal welfare matters for its own sake values animals with the capacity for welfare intrinsically to that extent).

We do not have enough data on these applied normative views to engage in any in-depth, direct, engagement with this debate in this paper. The participants in our research are engaged in animal tourism, and their views imply acceptance or endorsement of at least some of the fundamentals of some of these activities, most notably for present purposes animal tourism and associated conservation. However, this should not be taken as implying that the Māori ethical concepts and relationships we discuss do not suggest a normative approach to animal use, indeed, our discussion has covered many normative implications of Māori ethical concepts.

Most notably, Māori ethical concepts as they relate to animals found ethical regard for animals in a kinship relation—that of whakapapa. This relationship between humans and animals of mutual descent from the atua Papatūānuku and Ranginui means not just ethical duties to animals, but also a holistic identification, with all being part of the natural environment. This suggests a relational animal ethic.

Clare Palmer (2010) has advanced a relational ethic toward wild animals that is apposite to mention here [55]. We can only note some initial similarities and differences between the views of participants in this paper. Notably, Palmer’s relational ethic justifies an intuition that wild animals ought in many cases not to be assisted by people (i.e., where the requisite moral relations between people and animals are absent). We describe apparently similar endorsement of non-intervention to assist wild animals in some cases. However, we see evidence that this is based in a view that it is ultimately better for animals—the individual animal and the population as a whole, due to the holistic account of welfare we have described—if they are not assisted in those cases. Palmer rejects this view after finding counterexamples that show that human assistance would benefit the animal. It is notable for our purposes that Palmer considers only individualist accounts of welfare in her argument, which are the norm in Western philosophy of welfare. In the present paper the purported inconsistency that concerns Palmer is reconciled by the holistic linking of individual and group welfare that we describe, a view she does not consider. This reveals an alternative justification for a relational ethic that similarly supports the intuition that we often lack obligations to assist individual wild animals.

Tikanga Māori has also been characterized as a form of Aristotelian virtue theory [56]. The similarity that we describe of some aspects of mauri to the concept of *telos* found in virtue theory further supports this view. On this Aristotelian account, being moral is part of the *telos* of human life. When we are virtuous, we flourish—thus intimately linking human welfare with ethics. A virtue theoretic approach to animal use has potentially radical implications for current practice, particularly in farming, requiring farming practices and conditions be at least altered to allow animals to express their natures [57]. Although there is a virtue theory analysis of tourism, focusing on developing the moral character of tourists [58] its application to animals in tourism is so far absent [59]. We lack data here to do more than suggest that application and invite further research building on this.

### 5.2. Final Thoughts

This research is a preliminary study. There are limitations with the methodology as the field work was very much concentrated on local participants with iwi and hapū affiliations in the Otago/Southland region of Aotearoa New Zealand. It is not possible to generalize the findings, nevertheless the research provides a starting point for comprehending how Māori ethical concepts and values may inform and influence relationships with animals. This research represents the views of individuals from conservation and ecotourism backgrounds, further research is needed to represent the views of other stakeholders.

Informed by the interviews with Māori participants from particular iwi detailing how they value animals we have been able to identify important themes that align with views described in the literature [10,12,13,29]. Significant value was placed on native life that exceeded that of introduced species. Further, the well-being of these animals was thought to be linked to Māori concepts that are not present in the influential Western accounts of animal welfare we consider, although there may be some areas of compatibility nonetheless, as discussed. The majority of these concepts focused on the spiritual health and mauri of animals (and people as kaitiaki) in addition to their physical health. Finally, a holistic relationship between Māori and natural environments, including animals, was apparent. A lot of the care that goes into improving the welfare of animals is focused on the collective, landscape well-being rather than on distinct individuals. These views have both interesting similarities and differences to relational and virtue ethics as they apply to animals.

In New Zealand, the strengthening of Te Tiriti ō Waitangi relationships in conservation spaces has seen a valuing of mātauranga Māori and te ao Māori alongside Western ways of knowing, although much more progress is possible. Such ways of knowing contribute to our understanding of the connections between humans and animals, including, from an animal ethics context, what it means for an animal to live a good life and how animals ought to be treated.

This is, we hope, the beginning of a kōrero (conversation, discussion) about the welfare of animals, and our obligations to them, in this academic context. It is not, and not intended to be, a definitive account of these. This kōrero engages with one which is long and ongoing within te ao Māori, and we are very grateful to those within it who shared their time and knowledge with us.

## Data Availability

The data presented in this study may be available on request from the corresponding author. The data are not publicly available to protect anonymity of participants.
